# Beyond HIV prevention: Additional individual and community-level benefits of PrEP among Latino gay and bisexual men

**DOI:** 10.1371/journal.pone.0269688

**Published:** 2022-06-13

**Authors:** Ronald A. Brooks, Omar Nieto, Martin Santillan, Amanda Landrian, Anne E. Fehrenbacher, Alejandra Cabral

**Affiliations:** 1 Department of Family Medicine, University of California, Los Angeles, California, United States of America; 2 Center for HIV Identification, Prevention, and Treatment Services (CHIPTS), University of California, Los Angeles, California, United States of America; 3 Department of Research and Evaluation, Bienestar Human Services, Inc., Los Angeles, California, United States of America; 4 Department of Community Health Sciences, Fielding School of Public Health, University of California, Los Angeles, California, United States of America; 5 Division of Disease Prevention, Policy and Global Health, University of Southern California, Los Angeles, California, United States of America; Beth Israel Deaconess Medical Center/Harvard Medical School, UNITED STATES

## Abstract

**Background:**

HIV infections disproportionately impact Latino gay and bisexual men (GBM) in the United States. Pre-Exposure Prophylaxis (PrEP) is a proven prevention strategy that can help reduce new HIV infections in this population. Unfortunately, PrEP adoption and persistence among Latino GBM remain low. The added benefits of using PrEP experienced by Latino GBM can provide important insights to inform the development of PrEP messaging to motivate this population to explore and consider PrEP as an appropriate and acceptable HIV prevention tool.

**Methods:**

We conducted in-depth interviews with Latino GBM PrEP users to explore positive feelings and emotions, and additional benefits gained from using PrEP. Data were analyzed using thematic analysis.

**Results:**

A total of 29 Latino GBM completed the study interview. The average age of participants was 30 years, and the mean length of time using PrEP was 17.1 months. Five themes were constructed from the data representing the additional benefits gained by Latino GBM PrEP users, and included: (1) reduced fear, anxiety, and stress about HIV, HIV testing, and sex; (2) feeling empowered and in control of their HIV risk; (3) greater awareness of sexual risk behaviors and sexual health; (4) greater sexual exploration and pleasure, and comfort having condomless sex; and (5) a greater connection to community and a feeling of contributing to the elimination of HIV.

**Conclusions:**

The added benefits identified in this study represent a range of social, emotional, and psychological benefits that Latino GBM experience while using PrEP. They speak to the complementary benefits that PrEP can bring to Latino GBM who decide to use the medication, that go beyond HIV prevention. These findings can inform the development of future PrEP messaging to help improve motivation for PrEP uptake and persistent use among Latino GBM.

## Introduction

Latino gay and bisexual men (GBM) comprise one of the largest groups adversely affected by HIV in the United States. In 2017, at the time of this study, the rate of HIV diagnosis was 38% among Black GBM, 28% among Hispanic/Latino GBM, and 27% among White GBM [1]. As indicated in the technical notes within the CDC surveillance report, Hispanic/Latinos can be of any race (we use the term “Latino” in this manuscript) [2]. In addition, the CDC estimated that 1 in 2 Black GBM and 1 in 4 Latino GBM are projected to contract HIV in their lifetime compared to 1 in 11 for White GBM [3]. Further, while HIV diagnoses among Black and White GBM saw a period of stabilization from 2011 to 2015, diagnoses among Latino GBM did not see similar improvements [2]. To reduce new HIV infections among Latino GBM will require facilitating uptake of the most effective HIV prevention strategies, such as Pre-Exposure Prophylaxis (PrEP).

PrEP is a highly effective HIV prevention strategy with the ability to help curtail incident HIV infections among all at-risk populations [4]. As a result, PrEP has been listed as a key strategy in the *Ending the HIV Epidemic*: *A Plan for America initiative* [5]. Unfortunately, disparities persist in the adoption of PrEP among racial/ethnic sexual minority men, including Latino GBM [6]. Nationally, in 2017, only 31% of Latino GBM used PrEP in comparison to 42% of white GBM [6]. In California, a study in 2016 reported that only 6.6% of Latino GBM were using PrEP compared to 13.9% of white GBM [7]. In addition to suboptimal rates of adoption, there are also high rates of PrEP discontinuation in this population. In prior research among racial/ethnic sexual minority men, including Latino GBM, the median length of time using PrEP was only about 5 months [8, 9]. Further, a study surveying a racially and ethnically diverse sample of MSM and transgender women found that only about 38% of PrEP initiators continued using PrEP over a period of 12 months [10]. Facilitating greater uptake and persistence of PrEP may require moving beyond the biomedical focus on HIV prevention as a motivating factor for PrEP adoption, and, instead, highlighting other additional benefits of using PrEP for individuals and their community.

A primary benefit of PrEP is its effectiveness in preventing HIV infection. However, a growing body of research has identified additional benefits GBM receive from using PrEP, that go beyond HIV prevention, which include reduced anxiety and fear, improved sexual pleasure, and greater comfort with HIV-positive sex partners [11–22]. This research has been done with primarily White GBM and may not accurately reflect the experiences of Latino GBM. While some of benefits may be similar across all GBM populations, it is important to explore if these benefits are present among Latino GBM PrEP users, and if any unique benefits exist that could inform development of new types of messaging to facilitate greater PrEP adoption and persistence in this population. The present study addresses this gap in the existing research literature by uncovering the additional benefits of using PrEP specifically among Latino GBM PrEP users.

## Materials and methods

### Study overview

The LA [Los Angeles] PrEP Stories project was a qualitative study aimed at exploring the positive and negative experiences of using PrEP for HIV prevention among Latino and Black gay/bisexual men and other men who have sex with men. The focus of the study was on assessing the negative experiences resulting from anticipated, enacted, and internalized PrEP-related stigma. Those findings have been reported elsewhere [23–25]. A secondary focus was on investigating the positive experiences of using PrEP in the study population. For this article, we focus on the additional benefits of using PrEP experienced by Latino GBM. The added benefits of using PrEP among Black GBM is covered in the available literature [18, 26].

### Study population

Between January 2017 and October 2017, a purposive sample of Latino GBM PrEP users was recruited through sexual and social networking apps (i.e., Grindr and Growlr), community events, and community agency referrals to participate in an in-depth interview. Individuals were eligible to participate if they were 18 years of age or older, identified as Latino, identified as a man who has had anal sex with a male partner in the past six months, were currently taking Truvada® for PrEP, and resided in LAC.

### Data collection

A semi-structured interview guide was created to explore experiences of using PrEP in our study population. As part of the in-depth interview, we explored the positive experiences of using PrEP. Participants were asked to describe the feelings and emotions they experienced, and any added benefits gained from using PrEP. Interviews were conducted at a university-based research clinic by a trained interviewer. The interviewer’s characteristics reflected those of the study population (i.e., ethnicity, gender, sexual orientation, and PrEP use). Interviews were audio recorded and lasted 30 to 60 minutes. A brief survey was administered using Audio Computer-Assisted Self-Interview (ACASI) software to gather information on demographic and PrEP use characteristics. All interview audio files were transcribed verbatim and checked for accuracy by two research assistants. The Institutional Review Board of [Blinded for review] approved all study materials. All participants provided informed consent and received a $50 gift card for their participation.

### Data analysis

An inductive thematic analysis approach was used in analyzing the data [27]. Using this approach, we identified the added benefits from the participants’ reported experiences. The process began with the study team familiarizing itself with the data by conducting a line-by-line review of the transcripts and noting down any initial ideas. Initial codes were then generated using the interview guide, field notes, and the line-by-line review of transcripts, and included both descriptive and interpretive codes. Data analysis was performed by members of the study team, comprised of the study Principal Investigator (RAB), the project director/study interviewer (ON), and a doctoral student in Public Health (AL), all with years of experience analyzing qualitative data. The study team reviewed, discussed and modified the codes and their definitions, and identified exemplar quotes associated with each code before reaching consensus on the final codebook. In constructing themes, the team reviewed and clustered coded data that was similar and represented a meaningful pattern in the data. Themes were then reviewed in relation to the coded data to ensure that they represented the most important and relevant components of the data. ATLAS.ti (version 8.0.42), a qualitative data management and analysis software, was used in analyzing the data.

## Results

A total of 29 Latino GBM completed the study interview. Table 1 provides sociodemographic and PrEP use characteristics of the participants. The average age was 30 years (SD = 6.5; median = 29.8; range = 21–49). The vast majority identified as gay (86.2%) and reported completing at least some college (89.6%), working full or part-time (79.3%), and having an annual household income of $40,000 or less (79.2%). The mean length of time using PrEP was 17.1 months (SD = 16.2; median = 12.0; range = 0.25–68). Three-quarters (75.9%) self-reported “very good” or “excellent” PrEP medication adherence.

**Table 1:** Demographic and PrEP use characteristics (N = 29).

Variable	N (%) or M, SD
***Demographic Characteristics***	
Age (in years)	M = 29, SD = 6.5
Sexual Orientation	
Gay/homosexual/queer/same gender loving	25 (86.2)
Bisexual	4 (13.8)
Highest level of education completed	
High school graduate or received GED	3 (10.3)
Some college, AA degree, trade/technical school	13 (44.8)
Bachelor’s degree (BA, BS)	7 (24.1)
Some graduate school	2 (6.9)
Master’s degree	4 (13.8)
Employment status	
Working full-time	17 (58.6)
Working part-time	6 (20.7)
On permanent disability	1 (3.4)
Unemployed	5 (17.2)
Annual income	
$0–9,999	12 (54.5)
$10,000–19,999	2 (9.1)
$20,000–39,999	6 (27.3)
$40,000–59,999	2 (9.1)
$60,000–99,999	3 (10.3)
Health insurance	
Have health insurance	28 (96.6)
Does not have health insurance	1 (3.4)
***PrEP Use Characteristics***	
Length of time using PrEP (in months)	M = 17.1, SD = 16.2
Adherence to PrEP medication past month^1^	
Excellent	14 (48.3)
Very good	8 (27.6)
Good	5 (17.2)
Fair	2 (6.9)
Poor	0 (0)
Very poor	0 (0)

^1^PrEP adherence was measured via self-report using a validated Likert scale from excellent to very poor [28].

While HIV prevention was a primary motivator for starting PrEP among study participants, interviews with Latino GBM revealed additional benefits of using PrEP. From the data, we constructed five primary themes that represented these supplementary benefits: (1) reduced fear, anxiety, and stress about HIV, HIV testing, and sex; (2) feeling empowered and in control of their HIV risk; (3) greater awareness of sexual risk behaviors and sexual health; (4) greater sexual exploration and pleasure, and comfort having condomless sex; and (5) a greater connection to community and a feeling of contributing to the elimination of HIV. Exemplar quotes for each theme are presented and contextualized below. All names included in quotes are pseudonyms.

### Reduced fear, anxiety, and stress about HIV, HIV testing, and sex

One of the prominent benefits experienced by Latino GBM using PrEP is that it reduced the fear, anxiety, and stress associated with HIV, HIV testing, and sex. PrEP helped the men overcome the persistent fear they had about contracting HIV, as stated by Enrique, “*HIV has always been that dark cloud amongst young boy’s gay life*. *And really just like flying around like*, *‘When is it [HIV infection] going to hit me*, *when is it going to hit me*?*’ And now I don’t feel that*” (age 39, 37 months on PrEP). This fear was present even among participants who reported consistent condom use prior to initiating PrEP. Because of their PrEP use, the men also felt less anxiety about getting tested for HIV and receiving their test results, as captured by Carlos,

*“It is the guilt*. *It is a big thing on the back of your mind that you don’t have as much like when you’re going to get an HIV test…*. *You think*, *no*, *I’m perfectly protected…*. *I am already on PrEP*. *I am taking precautions for when they take that blood so it’s not going to turn a certain color [reactive]*. *So*, *it is a level of anxiety that you don’t have to worry about”* (age 24, 9 months on PrEP).

Participants also described how being on PrEP reduced their fear and anxiety about having sex and provided them with an improved state of calmness during sexual encounters, as expressed by Luis, *“I’m less nervous when having sex*. *Like*, *I’m not afraid anymore*. *I don’t stress about it like I would*. *It was terrible before*. *So*, *I’m just glad I don’t have to go through that anymore”* (age 24, 26.5 months on PrEP). In addition, this reduced fear and stress allowed some men to feel more comfortable about having sexual and/or romantic relationships with men who are living with HIV or of unknown HIV status, as stated by Enrique, “*I’m able to comfortably date somebody who’s HIV positive without a second thought*. *I’ve really embodied the whole ‘HIV-equal*,*’ as opposed to positive or negative because it doesn’t matter your status*” (age 39, 37 months on PrEP).

### Feeling empowered and in control of their HIV risk

The men described feeling empowered and more in control of their sex lives because of using PrEP. For example, participants described feeling more confident and having a more sex-positive attitude, as noted by Antonio, “*I think PrEP is very empowering and I think that’s what it does for me*, *and I would use that to be my message to other guys*. *Like*, *‘You can say fuck it and take the pill and be like*, *I like to fuck and I don’t want to worry about HIV*, *and being engaged in health care is going to keep me healthy*’” (age 32, 42 months on PrEP). Participants also described how PrEP gave them the power to protect themselves from HIV infection regardless of the behaviors or honesty of partners, as captured by Miguel:

“*I mean*, *it feels like I’m doing something*, *rather than relying on somebody else to do something because usually that’s how it is*. *If I’m the bottom*, *then I’m relying on somebody else to use the condom correctly and be honest with me about what their status is*. *Whereas with this*, *I mean*, *I still want them to be honest with me about their status*, *but I’m putting it on myself to take responsibility for protecting my health and that feels more empowering to me*” (age 37, 23 months on PrEP).

These feelings of personal control in preventing HIV infection cut across all types of sexual partners, from random hookups to regular partners.

### Greater awareness of sexual risk behaviors and sexual health

As a result of using PrEP, the men noted an increased self-awareness of their sexual risk-taking behaviors and sexual health. The men talked about being aware of the potential consequences of not using condoms consistently while on PrEP and how this behavior may result in a sexually transmitted infection (STI). However, STIs were viewed as treatable infections and the men were therefore more willing to take a calculated risk and not always use condoms. This sentiment is captured in this quote from Ricardo, “*Honestly*, *not every single time am I going to use a condom*…. *Yes*, *I can have chlamydia and that absolutely sucks*, *but that can get cured*. *I don’t know*, *there might be super chlamydia*, *but that’s a different conversation”* (age 21, 33 months on PrEP). They also described how PrEP provides protection when condoms fail or during periods when they decide not to use condoms, as narrated by Jorge, “*I think what keeps me from stopping being on PrEP is that if I’m not going to use condoms as often*, *at least I have PrEP*. *You know*. *And if I stop that [PrEP]*, *then that means that I have nothing*” (age 27, 18 months on PrEP). This sentiment was true for men who use condoms occasionally as well as for those men who disliked and never used condoms. Additionally, the men talked about a greater awareness of their general health as well as sexual health that came from using PrEP, as reported by Edgar:

*“Before I would get tested for HIV*, *like*, *once a year*. *Sometimes twice a year*. *STDs*, *probably like one or twice a year*. *Wherein now I’m testing every three months*, *and I think the more you’re keeping track of your health and where you’re at with STDs or your health and your liver functions*, *obviously because they check that for PrEP*, *you become more conscious”* (age 44, 27 months on PrEP).

### Greater sexual exploration and pleasure, and comfort having condomless sex

The men expressed how being on PrEP gave them more freedom and confidence for sexual exploration, facilitated greater sexual enjoyment, and made them feel more comfortable having condomless sex, if they so desired. The men narrated how prior to using PrEP there were certain sexual acts that they would not engage in for fear of contracting HIV (e.g., receptive anal sex), but noted how being on PrEP allowed them the freedom to explore sex more fully, as captured by these two participants:

*“I feel very sexually liberated*. *I’m able to do a lot of things*, *sexually*, *that I never thought I could do in my lifetime because of HIV and AIDS in the 80s and 90s when I was growing up”* (Enrique, age 39, 37 months on PrEP).

*“Not on PrEP*, *I didn’t feel comfortable doing a lot of stuff*, *but on PrEP*, *it seems to be*, *like*, *I’m freer in my decision making…*. *Like*, *I would not have receptive anal sex unprotected*, *not on PrEP*, *with someone who I wasn’t very confident was not HIV-positive*. *And so now I’m more willing to–that’s not an unacceptable risk”* (Miguel, age 37, 23 months on PrEP).

Being on PrEP also allowed the men to enjoy sex more because they felt protected, particularly during instances of possible exposure to HIV, as noted by Edgar, “*I enjoy sex more now because*, *you know*, *the times that my condom has broken*, *I’m not thinking*, *like*, *I’m at a major risk at all*” (age 44, 27 months on PrEP). Coupled with the greater sexual enjoyment was the comfort to have condomless sex, “*I feel more liberated*, *I guess*, *because we can have bareback sex more*,” noted Carlos (age 24, 9 months on PrEP). Using PrEP allowed the men to have the kind of sex life that they desired because of the absence of HIV-related stress.

### A greater connection to community and a feeling of contributing to the elimination of HIV

An additional benefit of using PrEP is that participants felt more connected to the communities they belong to, and that they were contributing to a reduction in new HIV infections through their own PrEP use and by encouraging others to use this prevention strategy. In constructing this theme, community was defined as the connection that a group has based on some common characteristic (e.g., PrEP users, gay/LGBTQ). In the narratives, the men speak of their connection to different communities. This greater sense of community connectedness and promotion of PrEP is captured in the quotes of these two participants:

*“I certainly feel more connected to the LGBT community in a certain way because I am using PrEP*. *I feel like I’m a part of a special club of gay men who are more conscious of their sex life and are more responsible”* (Julio, age 24, 4 months on PrEP).

*“The benefits that I’ve gained from using PrEP is being part of a larger movement and recognizing that me doing this [using PrEP] is hopefully*, *actively going to benefit the entire gay community*. *By me being on it [PrEP] and spreading the word of PrEP and spreading the message of how beneficial PrEP is and encouraging others to get on it*, *that hopefully they do the same and that this could be some sort of larger effect*, *where we now are more protected as a community*” (Ricardo, age 21, 33 months on PrEP).

Using PrEP gave participants a feeling of contributing to the elimination of HIV in the gay community, as described by Julio, “*It makes me feel like I am being more responsible*. *It makes me feel like I’m doing my part to eliminate HIV*” (age 24, 4 months on PrEP). In general, participants understood that having access to PrEP was an important step forward for the gay community, especially considering the impact the HIV epidemic continues to have on Latino GBM.

## Discussion

The added benefits of using PrEP identified in the present study represent a range of advantages that go beyond HIV prevention (e.g., reduced stress and anxiety, greater sexual exploration and enjoyment, feeling empowered). These additional positive effects of PrEP helped to improve the quality of the sexual lives of the Latino GBM in the study. While many of our findings align with prior research done with other populations of GBM [11–22], these findings provide the empirical evidence documenting the benefits of using PrEP exclusively in a population of Latino GBM living in a high HIV prevalence jurisdiction. In addition, we identified a unique benefit in our cohort of Latino GBM that has not been reported elsewhere: a feeling of greater connection to community and a sense that the men were helping to eradicate HIV. We expound on each benefit and explore how they can inform messaging in future PrEP campaigns and interventions to facilitate greater uptake and persistence among at-risk Latino GBM.

In our findings, Latino GBM described how PrEP helped reduce the fear, anxiety, and stress associated with contracting HIV, getting tested, and having sex. As described in the narratives, HIV represents “a dark cloud” that looms over the lives of Latino GBM, and HIV infection is perceived as an inevitable consequence for Latino GBM who are having sex with other men. However, PrEP helped to lift that dark cloud and provided Latino GBM with a sense of relief and reassurance that they will remain HIV uninfected. This newly acquired reduced stress and anxiety facilitated greater sexual experimentation and pleasure allowed the men to have the sex lives they desired. In agreement with other research [19, 29], we believe that PrEP promotional efforts can be improved by focusing on sexual pleasure in their messaging to Latino GBM. This type of messaging (e.g., one that highlights increased sexual fulfillment) may help motivate Latino GBM to initiate and persist on PrEP, particularly among those who experience persistent anxiety when having sex. It is important to find ways to remove the fear of sex and replace it with enjoyment.

The Latino GBM also described how PrEP made them feel empowered and in control of their sexual health. This occurred because the men no longer had to rely on their partners to protect them from HIV, thus reducing their vulnerability to potential exposure. In many ways, the introduction of PrEP changed the landscape of HIV prevention by offering GBM new opportunities for how they can explore their sexual lives. Prior to PrEP, HIV prevention options were limited and varied in efficacy (e.g., condom use, sero-sorting, and strategic positioning) [30–32]. To be effective, these strategies required trust and cooperation of a sexual partner. In contrast, PrEP alone is a highly effective prevention strategy, providing a level of sexual freedom that has not been available since before the onset of the HIV epidemic. Therefore, PrEP may be an optimal prevention method for Latino GBM who have difficulty negotiating safer sex practices with partners (e.g., asking about HIV status or requesting condoms be used). In this way, PrEP provides individuals with protection without them having to engage in sometimes uncomfortable and awkward conversations about HIV risk, HIV status, or condom use.

In addition, Latino GBM in this study had a greater consciousness and understanding of their HIV and STI risk behaviors because of the mandatory screenings for these infections required to maintain a PrEP prescription. This allowed some participants to feel comfortable taking calculated risks with their sexual partners (e.g., engaging in condomless sex). In addition, STIs were largely viewed as treatable infections. According to Tang et al. [33], the quarterly screenings required of all PrEP users play an important role in the early detection of STIs to prevent future infections among GBM. In addition, routine PrEP follow-up appointments keep Latino GBM engaged in medical care and that will hopefully translate to greater healthcare utilization for other health needs in this population.

A unique benefit identified in the present study was the reported feeling of a greater connection to community and of contributing to the elimination of HIV. The men spoke of community in different ways, sometimes referring to their connection to the broader gay/LGBTQ community and other times referring to the more intimate community of gay male PrEP users. This benefit was not noted in other research. Participants spoke of a camaraderie they had with other PrEP users and how being part of this community of PrEP users compelled them to actively disseminate PrEP information to peers in their community or social network in the hopes of increasing uptake. In effect, many of the participants became PrEP champions serving to educate, advocate, and promote PrEP [34]. This outcome highlights the significance of community-level diffusion in encouraging PrEP uptake in this population. In prior research, Page et al. (2017) noted the importance of involving community members and community-based organizations in promoting PrEP among Latino GBM. They described the use of *Promotores/as* (i.e., trusted community members) who work as health educators to disseminate information about PrEP. They also serve the role of peer navigators to help address barriers or concerns to initiation and retention in care. Page et al. (2017) further suggest the use of peer networks to promote information sharing [35]. These types of community activities will be important in promoting PrEP to Latino GBM as well as helping to destigmatize its use in the population at large.

The added benefits of using PrEP experienced by Latino GBM may help inform future PrEP promotion campaigns or interventions targeting this population. As a way of motivating Latino GBM to use PrEP, future messaging targeting this population should highlight the supplementary positive effects PrEP affords users that go beyond the strictly biomedical focus on HIV prevention. Overall, the added benefits identified in the present study speak to a reduced fear, anxiety, and stress associated with HIV and having sex, as well as greater sexual exploration and enjoyment. As such, PrEP messaging to Latino GBM should focus on providing men the opportunity to have the sex life they desire. Drawing from the narratives of the Latino GBM in the study, we offer the following suggestions of possible messages that would resonate with the population (i.e., “I’m having more fun now than I ever had before, because I don’t live with fear.,” “It’s okay for you to be attracted to other men. Here’s this pill that will take away a lot of fear and anxiety.” “I’m able to have more fun without worrying about HIV.” “I enjoy sex more now.” “When I’m having sex, I’m enjoying the moment.” “Mentally and emotionally, it gives me peace of mind.” “I feel more in control.” “I’m doing something, rather than relying on somebody else to [protect me from HIV].” “It’s made me worry less about HIV.” “I’m less nervous when having sex.” “It allows me to have the sex life that I want.”). These messages can be delivered in the form of personal testimonials of Latino GBM PrEP users. They can be used in PrEP campaigns, shared on social media platforms of community-based organizations or PrEP programs serving Latino GBM, or used by local public health departments in PrEP campaigns seeking to target this population.

Another important component of PrEP messaging to Latino GBM should center on the connection to community and helping to eradicate HIV in the community. Drawing on a quote from a study participant, a potential message can center on Latino GBM contributing to eradication of HIV in the community (e.g., “I’m doing my part to eliminate HIV.”). A community-level approach to PrEP promotion may resonate with Latino GBM, in particular, given the collectivist nature of Latino culture [36]. In a collectivist culture, group activities are dominant, responsibility is not individually based but shared, and accountability is collective [37]. In addition, as noted in prior work, among Latinos, the individual is subordinate to the group (i.e., the “we” takes precedence over “I”) [38]. As such, new PrEP promotion efforts may be more effective if they draw on community members (e.g., Latino LGBT community in particular) to motivate Latino GBM to use PrEP in support of a healthier community.

These findings should be interpreted in consideration of the study limitations. Our sample was recruited in Los Angeles County with a large Latino population, and, therefore, results may not reflect the experiences of Latino GBM PrEP users in other settings (e.g., rural settings with smaller Latino populations). In addition, the sample consists exclusively of English-speaking Latino GBM and may not reflect the experiences of Spanish-speaking Latino GBM PrEP users. Research with monolingual Spanish-speaking Latino GBM is needed to assess if positive experiences and benefits gained from PrEP use differ based on language and other related cultural nuances associated with monolingual Spanish-speaking populations. A final limitation is that the data were collected in 2017 and uptake has changed since the study took place. A 2020 CDC report noted that there has been an increase in PrEP use across all racial/ethnic groups of 25%; however, uptake remains lower among Latinos (16%) compared to Whites (66%) [39].

## Conclusion

Overall, these findings highlight important positive attributes of PrEP that Latino GBM experience that go beyond protection from HIV infection. They address important components in the sexual lives of Latino GBM and can help inform the messaging and promotion of PrEP to this population. While it remains essential to address existing systemic and social barriers to PrEP (e.g., cost, access, homophobia, stigma), these findings offer opportunities for new approaches to motivate Latino GBM to explore and consider PrEP as an appropriate and acceptable HIV prevention tool.

## Supporting information

S1 Appendix (DOCX)

## References

[1] Centers for Disease Control and Prevention. HIV Surveillance Report, 2017. [Internet]. 2018. Available from: https://www.cdc.gov/hiv/pdf/library/reports/surveillance/cdc-hiv-surveillance-report-2017-vol-29.pdf

[2] Centers for Disease Control and Prevention. Estimated HIV incidence and prevalence in the United States, 2015–2019. 2021;26(1):81.

[3] Centers for Disease Control and Prevention. 2016 CROI Press Release: Lifetime HIV Risk [Internet]. 2018 [cited 2022 May 5]. Available from: https://www.cdc.gov/nchhstp/newsroom/2016/croi-press-release-risk.html

[4] Spinner CD, Boesecke C, Zink A, Jessen H, Stellbrink HJ, Rockstroh JK, et al. HIV pre-exposure prophylaxis (PrEP): a review of current knowledge of oral systemic HIV PrEP in humans. Infection. 2016 Apr 1;44(2):151–8. doi: 10.1007/s15010-015-0850-2 26471511

[5] HIV.gov. What is ‘Ending the HIV Epidemic: A Plan for America’? [Internet]. HIV.gov. 2020 [cited 2021 Oct 4]. Available from: https://www.hiv.gov/federal-response/ending-the-hiv-epidemic/overview

[6] Pitasi MA, Beer L, Cha S, Lyons SJ, Hernandez AL, Prejean J, et al. Vital Signs: HIV Infection, Diagnosis, Treatment, and Prevention Among Gay, Bisexual, and Other Men Who Have Sex with Men—United States, 2010–2019. MMWR Morb Mortal Wkly Rep. 2021 Dec 3;70(48):1669–75. doi: 10.15585/mmwr.mm7048e1 34855721 PMC8641567

[7] Pulpisher C, Montoya J, Curtis P, Holloway IW, Leibowitz A. Addressing PrEP Disparities among Young Gay and Bisexual Men in California [Internet]. 2016. Available from: https://aplahealth.org/wp-content/uploads/2016/09/APLA_PrEP_FullReport_WEB.pdf

[8] Liu A, Coleman K, Walker N, Vittinghoff E, Turner C, Vinson J, et al. Assessing the PrEP Continuum in the San Francisco Bay Area: The Quickie Mobile Survey. In: Conference on Retroviruses and Opportunistic Infections (CROI)Conference on Retroviruses and Opportunistic Infections (CROI)Conference on Retroviruses and Opportunistic Infections (CROI) [Internet]. Seattle, WA; 2019 [cited 2021 Sep 15]. Available from: https://www.croiconference.org/abstract/assessing-prep-continuum-san-francisco-bay-area-quickie-mobile-survey/

[9] Rao DW, Carr J, Naismith K, Hood JE, Hughes JP, Morris M, et al. Monitoring HIV Preexposure Prophylaxis Use Among Men Who Have Sex With Men in Washington State: Findings From an Internet-Based Survey. Sex Transm Dis. 2019 Apr;46(4):221–8. doi: 10.1097/OLQ.0000000000000965 30870322 PMC7687921

[10] Spinelli MA, Scott HM, Vittinghoff E, Liu AY, Gonzalez R, Morehead-Gee A, et al. Missed Visits Associated With Future Preexposure Prophylaxis (PrEP) Discontinuation Among PrEP Users in a Municipal Primary Care Health Network. Open Forum Infect Dis. 2019 Apr;6(4):ofz101. 10.1093/ofid/ofz101 PMC6441570 30949540

[11] Grant RM, Koester KA. What People Want From Sex and PrEP. Curr Opin HIV AIDS. 2016 Jan;11(1):3–9. doi: 10.1097/COH.0000000000000216 26569183 PMC6446930

[12] Grov C, Westmoreland DA, D’Angelo AB, Pantalone DW. How Has HIV Pre-Exposure Prophylaxis (PrEP) Changed Sex? A Review of Research in a New Era of Bio-behavioral HIV Prevention. J Sex Res. 2021 Sep;58(7):891–913. doi: 10.1080/00224499.2021.1936440 34180743 PMC9729849

[13] Haire B, Murphy D, Maher L, Zablotska-Manos I, Vaccher S, Kaldor J. What does PrEP mean for ‘safe sex’ norms? A qualitative study. PLOS ONE. 2021 Aug 5;16(8):e0255731. doi: 10.1371/journal.pone.0255731 34352034 PMC8341650

[14] Hammack PL, Toolis EE, Wilson BDM, Clark RC, Frost DM. Making Meaning of the Impact of Pre-Exposure Prophylaxis (PrEP) on Public Health and Sexual Culture: Narratives of Three Generations of Gay and Bisexual Men. Arch Sex Behav. 2019 May;48(4):1041–58. doi: 10.1007/s10508-019-1417-6 30874978 PMC7175836

[15] Keen P, Hammoud MA, Bourne A, Bavinton BR, Holt M, Vaccher S, et al. Use of HIV Pre-exposure Prophylaxis (PrEP) Associated With Lower HIV Anxiety Among Gay and Bisexual Men in Australia Who Are at High Risk of HIV Infection: Results From the Flux Study. J Acquir Immune Defic Syndr 1999. 2020 Feb 1;83(2):119–25. doi: 10.1097/QAI.0000000000002232 31935203

[16] Koester K, Amico RK, Gilmore H, Liu A, McMahan V, Mayer K, et al. Risk, safety and sex among male PrEP users: time for a new understanding. Cult Health Sex. 2017 Dec;19(12):1301–13. doi: 10.1080/13691058.2017.1310927 28415911

[17] Pantalone DW, Holloway IW, Goldblatt AEA, Gorman KR, Herbitter C, Grov C. The Impact of Pre-Exposure Prophylaxis on Sexual Communication and Sexual Behavior of Urban Gay and Bisexual Men. Arch Sex Behav. 2020 Jan;49(1):147–60. doi: 10.1007/s10508-019-01478-z 31628628 PMC7018565

[18] Quinn KG, Christenson E, Sawkin MT, Hacker E, Walsh JL. The Unanticipated Benefits of PrEP for Young Black Gay, Bisexual, and Other Men Who Have Sex with Men. AIDS Behav. 2020 May;24(5):1376–88. doi: 10.1007/s10461-019-02747-7 31768688 PMC7592111

[19] Van Dijk M, De Wit JBF, Guadamuz TE, Martinez JE, Jonas KJ. Quality of Sex Life and Perceived Sexual Pleasure of PrEP Users in the Netherlands. J Sex Res. 2021 Jun 15;1–6. doi: 10.1080/00224499.2021.1931653 34128741

[20] Whitfield THF, Jones SS, Wachman M, Grov C, Parsons JT, Rendina HJ. The Impact of Pre-Exposure Prophylaxis (PrEP) Use on Sexual Anxiety, Satisfaction, and Esteem Among Gay and Bisexual Men. J Sex Res. 2019 Dec;56(9):1128–35. doi: 10.1080/00224499.2019.1572064 30777781 PMC6699935

[21] Yang C, Krishnan N, Kelley E, Dawkins J, Akolo O, Redd R, et al. Beyond HIV prevention: a qualitative study of patient-reported outcomes of PrEP among MSM patients in two public STD clinics in Baltimore. AIDS Care. 2020 Feb;32(2):238–41. doi: 10.1080/09540121.2019.1622639 31146549 PMC6884665

[22] Zapata JP, Petroll A, de St Aubin E, Quinn K. Perspectives on Social Support and Stigma in PrEP-related Care among Gay and Bisexual Men: A Qualitative Investigation. J Homosex. 2020 Sep 22;1–23. doi: 10.1080/00918369.2020.1819709 32960750

[23] Brooks RA, Landrian A, Nieto O, Fehrenbacher A. Experiences of Anticipated and Enacted Pre-exposure Prophylaxis (PrEP) Stigma Among Latino MSM in Los Angeles. AIDS Behav. 2019 Jul 1;23(7):1964–73. doi: 10.1007/s10461-019-02397-9 30649635 PMC6571053

[24] Brooks RA, Nieto O, Landrian A, Fehrenbacher A, Cabral A. Experiences of Pre-Exposure Prophylaxis (PrEP)–Related Stigma among Black MSM PrEP Users in Los Angeles. J Urban Health [Internet]. 2019 Jun 18 [cited 2019 Aug 23]; Available from: http://link.springer.com/10.1007/s11524-019-00371-3 10.1007/s11524-019-00371-3 PMC7560642 31214977

[25] Brooks RA, Cabral A, Nieto O, Fehrenbacher A, Landrian A. Experiences of Pre-Exposure Prophylaxis Stigma, Social Support, and Information Dissemination Among Black and Latina Transgender Women Who Are Using Pre-Exposure Prophylaxis. Transgender Health. 2019 Aug 30;4(1):188–96. doi: 10.1089/trgh.2019.0014 31482134 PMC6716188

[26] Huang W, Lockard A, Kelley CF, Serota DP, Rolle CPM, Sullivan PS, et al. From declining PrEP to PrEP initiation as “first nature”—what changes PrEP initiation decisions among young, Black MSM. AIDS Care. 2022 Mar;34(3):284–93. doi: 10.1080/09540121.2021.1960946 34369230 PMC8825883

[27] Braun V, Clarke V. Thematic analysis. In: APA handbook of research methods in psychology, Vol 2: Research designs: Quantitative, qualitative, neuropsychological, and biological. Washington, DC, US: American Psychological Association; 2012. p. 57–71. (APA handbooks in psychology®).

[28] Feldman BJ, Fredericksen RJ, Crane PK, Safren SA, Mugavero MJ, Willig JH, et al. Evaluation of the Single-Item Self-Rating Adherence Scale for Use in Routine Clinical Care of People Living with HIV. AIDS Behav. 2013 Jan 1;17(1):307–18. doi: 10.1007/s10461-012-0326-7 23108721 PMC3549002

[29] Mabire X, Puppo C, Morel S, Mora M, Rojas Castro D, Chas J, et al. Pleasure and PrEP: Pleasure-Seeking Plays a Role in Prevention Choices and Could Lead to PrEP Initiation. Am J Mens Health. 2019 Feb;13(1):1557988319827396. 10.1177/1557988319827396 PMC6440035 30819060

[30] Eaton L, Kalichman S, O’Connell D, Karchner W. A strategy for selecting sexual partners believed to pose little/no risks for HIV: Serosorting and its implications for HIV transmission. AIDS Care. 2009 Oct 1;21:1279–88. doi: 10.1080/09540120902803208 20024704 PMC2937200

[31] Gafos M, Horne R, Nutland W, Bell G, Rae C, Wayal S, et al. The Context of Sexual Risk Behaviour Among Men Who Have Sex with Men Seeking PrEP, and the Impact of PrEP on Sexual Behaviour. AIDS Behav. 2019 Jul;23(7):1708–20. doi: 10.1007/s10461-018-2300-5 30306439 PMC6570678

[32] Van de Ven P, Kippax S, Crawford J, Rawstorne P, Prestage G, Grulich A, et al. In a minority of gay men, sexual risk practice indicates strategic positioning for perceived risk reduction rather than unbridled sex. AIDS Care. 2002 Aug;14(4):471–80. doi: 10.1080/09540120208629666 12204150

[33] Tang EC, Vittinghoff E, Philip SS, Doblecki-Lewis S, Bacon O, Chege W, et al. Quarterly screening optimizes detection of sexually transmitted infections when prescribing HIV preexposure prophylaxis. AIDS Lond Engl. 2020 Jul 1;34(8):1181–6. doi: 10.1097/QAD.0000000000002522 32205724

[34] Brooks RA, Nieto O, Landrian A, Fehrenbacher A, Cabral A. Experiences of Pre-Exposure Prophylaxis (PrEP)-Related Stigma among Black MSM PrEP Users in Los Angeles. J Urban Health Bull N Y Acad Med. 2020 Oct;97(5):679–91. doi: 10.1007/s11524-019-00371-3 31214977 PMC7560642

[35] Page KR, Martinez O, Nieves-Lugo K, Zea MC, Grieb SD, Yamanis TJ, et al. Promoting Pre-exposure Prophylaxis to Prevent HIV Infections Among Sexual and Gender Minority Hispanics/Latinxs. AIDS Educ Prev Off Publ Int Soc AIDS Educ. 2017 Oct;29(5):389–400. doi: 10.1521/aeap.2017.29.5.389 29068715 PMC5765546

[36] Elder JP, Ayala GX, Parra-Medina D, Talavera GA. Health communication in the Latino community: issues and approaches. Annu Rev Public Health. 2009;30:227–51. doi: 10.1146/annurev.publhealth.031308.100300 19296776

[37] Gudykunst WB. Bridging differences: Effective intergroup communication, 3rd ed. Thousand Oaks, CA, US: Sage Publications, Inc; 1998. xv, 351 p. (Bridging differences: Effective intergroup communication, 3rd ed).

[38] Ramirez-Valles J. The Quest for Effective HIV-Prevention Interventions for Latino Gay Men. Am J Prev Med. 2007 Apr 1;32(4):34–5. doi: 10.1016/j.amepre.2006.12.024 17386334

[39] Centers for Disease Control and Prevention. Core indicators for monitoring the Ending the HIV Epidemic initiative (preliminary data): National HIV Surveillance System data reported through June 2021; and preexposure prophylaxis (PrEP) data reported through March 2021 [Internet]. [cited 2022 May 13]. Available from: https://www.cdc.gov/hiv/pdf/library/reports/surveillance-data-tables/vol-2-no-4/cdc-hiv-surveillance-tables-vol-2-no-4.pdf

