# Supplementary material for: Beyond HIV prevention: Additional individual and community-level benefits of PrEP among Latino gay and bisexual men
Source: PLoS One. 2022 Jun 13;17(6):e0269688. doi: 10.1371/journal.pone.0269688 (PMC9191695; doi:10.1371/journal.pone.0269688)
Supplement: S1 Appendix — (DOCX) [file pone.0269688.s001.docx]

**Interview Guide**

**LMSM PrEP user interview**

1. When did you first start taking PrEP, the approximate date of when you started?
2. What was the main reason you decided to use PrEP?
3. We talked about some of your reasons for starting PrEP. Now I want to focus on how you feel about PrEP. So, what are some of your personal feelings about PrEP?

Probes:

- 1. For example, have you ever felt embarrassed because of your PrEP use? If yes, please describe why you felt embarrassed.
  2. Have you ever felt proud that you are using PrEP? If yes, please describe why you felt proud.
  3. Have you ever felt guilty because of your PrEP use? If yes, please describe why you felt guilty.
  4. Have you felt more responsible for your PrEP use? If yes, please describe why you felt more responsible.
  5. Does using PrEP make you feel less anxious? If yes, please describe why you feel less anxious?
  6. Does using PrEP ever make you feel like you are doing something wrong? If yes, please describe why you feel like you are doing something wrong.

1. Can you describe any of the benefits you have gained from using PrEP?
2. Has PrEP changed your condom use?

Probes:

- 1. If no, how would you describe your condom use?
  2. If yes, how has your condom use changed?

1. Do you have sex with more partners now that you’re on PrEP?
2. What do [Latino/Black] gay and bisexual men need to know about PrEP in order to decide if it’s right for them (e.g., finding a doctor that prescribes PrEP, side effects, effectiveness)?
3. What will motivate [Latino/Black] gay and bisexual men to use PrEP?
4. Do you have any closing comments that you would like to make about your experiences using PrEP or about PrEP in general?
